# Metabolic Effects of Fasting and Animal Source Food Avoidance in an Ethiopian Adult Cohort

**DOI:** 10.1038/s41598-019-53185-3

**Published:** 2019-11-18

**Authors:** Makeda Sinaga, Melese Sinaga Teshome, Radiet Kidane, Tilahun Yemane, Elsah Tegene, David Lindstrom, Tefera Belachew

**Affiliations:** 1 Department of Nutrition and Dietetics, Jimma University, Jimma, South West Ethiopia; 2 Department of Laboratory Sciences, Jimma University, Jimma, South West Ethiopia; 3 Department of Internal Medicine, Jimma University, Jimma, South West Ethiopia; 4 Population Studies Centre, Brown University, Providence, USA

**Keywords:** Health services, Nutrition, Epidemiology

## Abstract

Fasting is a religious practice to which the faithful comply strictly. The longest period of fasting in Orthodox religion is the lent (in Ethiopia known as “*Hudade”*). According to the doctrine of Ethiopian Orthodox Christianity, fasters should strictly avoid all animal source foods (ASF) and skip breakfast at least up to lunch time. This can be taken as a well-controlled natural experiment to evaluate the effect of breakfast skipping and avoidance of ASF for 55 days. However, there is no study that evaluated the effect of ASF fasting (avoidance of animal source foods  and breakfast skipping) on lipid profiles, fasting blood sugar and body composition in Ethiopian set up. A retrospective cohort study was carried out among 704 employees of Jimma University (253 fasters and 451 non-fasters) from February 2015 to April 2015. Data on socio-demographic, anthropometry, blood pressure and blood samples were collected according to WHO STEPS procedure. Descriptive statistics and multivariable linear regression models were used to compare the effect of fasting on outcome variables. There was a significant difference in the body fat percent (mean ± sd) between non-fasters (32.35 ± 11.12) and fasters (30.59 ± 11.22, *P* = *0.045*). Similarly, the mean ± sd waist circumference was higher among non-fasters (84.96 ± 11.43 cm) compared to fasters (83.04 ± 11.43 cm, *P* < *0.033*). High density lipoprotein was significantly (*P* = *0.001*) high among fasters (68.29 mg/dl) compared to non-fasters (57.24 mg/dl). Total cholesterol (T.chol) was also higher among non- fasters (181.01 mg/dl) than fasters (173.80 mg/dl, *P* = *0.035*). The mean Triglyceride level was significantly (*P* = *0.035)* high among non-fasters (142.76 mg/dl) compared to fasters (129.39 mg/dl). Similarly, fasting blood sugar was high among non-fasters (100.14 mg/dl) compared to fasters (95.11 mg/dl), *P* = *0.009*. On multivariable linear regression analyses after adjusting for different variables, fasters had a significantly high mean HDL and lower mean T.chol, Triglycerides, FBS and LDL levels. Similarly, fasters had a significantly low mean waist circumference and low mean body fat percent (*P* < *0.05*). In conclusion, animal source food avoidanceand breakfast skipping has a significant desirable health effects on lipid profiles, fasting blood sugar and body composition. The findings imply the need for considering such a dietary practice as a basis for public health promotion. Future research should investigate the effect of ASF fasting and breakfast skipping on micronutrient intake and determine the minimum number of days of fasting required to generate clinically significant effects.

## Introduction

Fasting defined as abstinence from food for varying duration, has been associated with increased longevity and improved human health^1^. Although fasting is practiced for various reasons from time immemorial, its role in optimizing energy metabolism and strengthening cellular protection was elucidated since recent past^2–4^. Animal studies showed that intermittent or periodic fasting reduced the risk of diabetes, cancer, heart disease, and neurodegeneration^3^. Similarly, alternate day fasting in human beings also showed reductions in body weight accompanied by lower blood pressure, low density lipoprotein (LDL), triglycerides (TG), and insulin resistance^5^.

In some countries, Orthodox Christianity fasting includes avoidance of meat, fish, dairy products and olive oil mimicking the Mediterranean diet^1^. During fasting days, people follow a vegetarian diet, which is associated with low prevalence of cardiovascular disease and longevity^6^. A study on the effect of Greek Orthodox regular fasting demonstrated that fasting lead to a non-significant reduction in high density lipoprotein (HDL) and possible impact on obesity^7^.

Avoidance of animal source foods during fasting will cut-down animal fat intake. Promotion of vegetarian diet (avoiding  animal source food) has been used as an intervention to reduce the prevalence of metabolic syndrome (MetS). Studies in different parts of the world showed that having vegetarian dietary pattern is associated with more favorable profile of metabolic risk factors and lower risk of MetS^8–11^. It was also reported that composite diets such as DASH diet, Mediterranean diet, and ‘prudent’ diet have been documented to reduce chronic diseases^12^. The Mediterranean style diet was significantly associated with lower body mass index (BMI), fat mass, glucose, T.chol, TG, HDL and LDL levels^13^.

However, the vegan (absolute vegetarian) diet did not decrease the risk of MetS compared with pescovegetarian, lactovegetarian and no vegetarian diet in Taiwan^14^. Saturated fats from animals and transfats are incriminated to be the main causes of metabolic syndrome^12^. A study on obese individuals showed that intermittent fasting reduced oxidative stress^2^. Breakfast skipping was shown to have an impact on selected metabolic parameters, nutrient intakes and body weight in university going adults^15^. A study on the effect of breakfast skipping on healthy Korean adults showed that eating breakfast regularly enhances dietary quality, but may increase the risk of elevated serum triglycerides^16^.

Ethiopian Orthodox Christianity instructs its followers to fast during the lent, traditionally known as “Hudade Tsom”, by avoiding all animal source foods and skipping breakfast at minimum up to 12:00 PM or until 15:00 PM as far as possible for duration of 55 days^17^.

So far, there is no study that documented the effect of such fasting practice on components of metabolic syndrome, lipid profile and body composition. Exploration of the effect of this natural phenomenon on the markers of metabolic syndrome and body composition can give clues to design future interventions. It will help to answer different questions including: Is avoiding animal source food and skipping breakfast associated with level of lipid profile? with anthropometric markers of metabolic syndrome?

## Methods

### Study participants and setting

A total of 704 participants (253 fasters and 451 non-fasters) were randomly selected from employees of Jimma University using their payroll as a sampling frame. A retrospective cohort study was employed to compare fasters and non-fasters during the lent fasting of Ethiopian Orthodox Christians. Known cases of diabetes and hypertension were excluded from the study as they are taking medications which may interfere with their fasting status.

### Measurements

Data collection was started after 40 days of “Hudade” fasting and continued up to 55 days. Both fasters and non-fasters were retrospectively interviewed for potential predictor variables including physical activity, sleep pattern, alcohol intake and sedentary behavior. For this study, data collected for the development of optimal anthropometric cut-off for diagnosing obesity among Ethiopian adults were used. The details of the methods including the sample size, sampling methods, inclusion and exclusion criteria, are described elsewhere^18^.

Household income was rank ordered into tertiles; while education level was classified into three categories: high school and below, diploma and degree and above. Similarly hours spent sitting/reclining was also rank ordered into tertiles.

#### Weight, height and body composition

Weight and height were measured with participants wearing light clothing and no shoes. Weight was measured with a digital scale (Model 871, Seca, Germany) accurate to 100 grams. Height was measured with an adjustable portable stadiometer which was accurate to 0.1 cm with four points (heel, calf, buttocks and shoulders) touching the vertical stand of the stadiometer and head adjusted to Frankfurt plane. Standardization exercise was performed during the training of anthropometry measurers. Body Mass Index (BMI) was calculated as weight in kilograms (kg) divided by height in meters squared (m^2^). Waist circumference was measured at the midway between the lowest costal margin at the level of mid clavicular line and anterior superior iliac spine using a non-stretchable measuring tape. Body composition was determined using different anthropometric indices BMI, Waist Circumference (WC) and body fat percent measured using Air Displacement Plethysmography (ADP). All anthropometric measurements were taken in the morning after an overnight fasting.

### Lipid profiles and fasting blood sugar

To determine lipid profiles and fasting blood sugar levels, five ml of venous blood was collected after an overnight fasting. Fasting blood sugar was determined using Humastar within two hours of collection in Jimma University Specialized Hospital (JUSH) at JUCAN project laboratory. Lipid profiles (high density lipoprotein, total cholesterol and triglycerides) were determined from the serum using Humastar 80 machine in star III laboratory of Mettu Karl Hospital following standard operating procedures as described elsewhere^18^.

#### Sleep duration

Was determined based on responses to the question, “…how many hours do you usually sleep undisturbed each day?” Participants responded by reporting the number of hours of undisturbed sleep they usually had.

#### Animal source food avoidance and breakfast skipping

Breakfast skipping was measured by asking the following question to the fasters during the lent: “how do you fast?” All the fasters reported that they avoided ASF totally and some of them skipped breakfast up to lunch time; while others fasted until 15:00 pm. The study participants were categorized as fasters based on self-reported avoidance all animal source foods, and skipping breakfast at least up to lunch time (12:00 AM), while those not fulfilling these two conditions were defined as non-fasters.

### Ethical approval and consent to participate

Ethical clearance was obtained from Jimma University Institutional Review Board (IRB). Clinical directors, administration office and collage deans were informed about the study objectives through letter written from Jimma University IRB office to enhance cooperation. All data collection procedures were performed in accordance with the relevant guidelines and regulations of Jimma University and Ethiopian National Research Ethics. Informed written consent was taken from each selected participant to confirm willingness after explanation of the survey purpose and its benefits. The study participants were assured that they are free to withdraw their consent and discontinue participation in the study without any form of prejudice. Privacy and confidentiality of the collected data were ensured throughout the study. All study participants were given the results of their blood sugar, blood pressure, body fat percent and lipid profile and those with problems were given education and instructions to visit a doctor.

## Results

### Background characteristics

A total of 704 employees of Jimma University (253 fasters and 451 non-fasters) participated in the study. Table 1 presents the background characteristics of the study participants by their fasting status. The mean ± SD age of study participants was 35.27(±9.77) years for non-fasters and 36.23(±9.58) years for fasters. The proportion of females was 49.9% for fasters and 67.6% for non-fasters.

**Table 1:** Background characteristics of Jimma University employees involved in the study.

Variables	Fasting		P
	No	Yes	
	n (%)	n (%)	
Sex			
Female	225 (49.9)	171 (67.6)	0.001
Male	226 (50.1)	82 (32.4)	
Age (Yrs), Mean ± (sd)	35.27 (9.8)	36.23 (9.6)	0.096^a^
Educational status			
High school and below	170 (37.7)	135 (53.4)	
Diploma	119 (26.4)	53 (20.9)	<0.001
Degree and above	162 (35.9)	65 (25.7)	
Type of staff			
Administrative staff	308 (68.3)	204 (80.6)	
Academic staff	120 (26.6)	39 (15.4)	
Hospital Technical staff	23 (5.1)	10 (4.0)	0.002
Monthly Income Tertile			
High	173 (38.4)	61 (24.1)	
Medium	149 (33.0)	85 (33.6)	<0.001
Low	129 (28.6)	107 (42.3)	
Frequency of Alcohol Drinking			
Non-drinker or Less than once per month	357 (79.2)	178 (70.4)	0.009
1–3 days per month or more	94 (20.8)	75 (29.6)	
Number of days of moderate intensity exercise per week			
No or Less than 3 days per week	348 (77.2)	207 (81.8)	0.147
Three or more days per week	103 (22.8)	46 (18.2)	
Number hour spent sitting per day (mean ± sd)	5.69 (7.8)	4.97 (6.0)	0.081^a^
Number of hours of sleep per night (mean ± sd)	7.22 (1.5)	7.82 (5.4)	0.038^a^

Fasting = avoiding animal; source food (ASF) and Breakfast skipping until 12:00 AM or 15:00 PM.

^a^Mann- Whitney U test.

Among the study participants, 79.2% of non-fasters and 70.4% of the fasters did not drink alcohol or drunk less than once per month. The mean (±sd) hours slept per night were 7.22(±1.47) and 7.82(±5.39) for non-fasters and fasters, respectively.

### Differences in body composition, blood sugar and lipid profiles

The mean difference in body composition, fasting blood sugar and lipid profiles between fasters and non-fasters is shown in Table 2. Fasters had a significantly (*P* = *0.045)* low mean body fat percent and waist circumference (*P* < *0.033)* compared to non-fasters. Similarly, fasters had a significantly low mean T.chol (*P* = *0.035)*, Triglyceride (*P* = *0.035*), low density lipoprotein (P = 0.015) and fasting blood sugar (*P* = *0.009*; while they had a significantly higher mean high density lipoprotein (*P* = *0.001)* compared to non-fasters.

**Table 2:** Differences in body composition, fasting blood sugar and lipid profiles between fasters and non-faster employees of Jimma University.

	Fasting	N	Mean	Sd.	*P*
Body fat percent	Non Faster	451	32.35	11.12	*0.045*
	Faster	253	30.59	11.22	
Body mass index (Kg/m^2^)	Non Faster	451	24.00	4.81	*0.669*
	Faster	253	24.16	4.88	
Waist Circumference (cm)	Non Faster	451	84.96	11.43	*0.033*
	Faster	253	83.04	11.43	
High Density Lipoprotein (mg/dl)	Non Faster	451	57.24	39.22	*0.001*
	Faster	253	68.29	47.37	
Cholesterol (mg/dl)	Non Faster	451	181.01	53.63	*0.035*
	Faster	253	173.80	36.45	
Low Density Lipoprotein (LDL) mg/dl)	Non Faster	451	97.98	42.90	0.015
	Faster	253	89.55	45.40	
Triglyceride (mg/dl)	Non Faster	451	142.76	85.62	0.035
	Faster	253	129.39	77.57	
Fasting Blood sugar (mg/dl)	Non Faster	451	100.14	33.46	*0.009*
	Faster	253	95.11	17.73	

Sd = Standard deviation, Mg/dl = milligram/deciliter.

**Figure 1:**
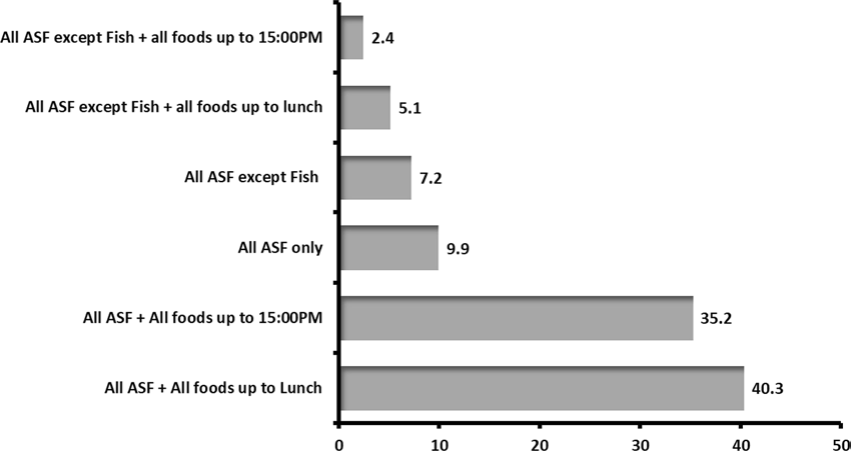
Types of fasting among employees of Jimma University, Southwest Ethiopia.

Among fasters, large proportion fasted all ASF and all foods up to lunch time (40.3%) followed by those who fasted all ASF and all foods up to 15:00 PM (35.2%). The remainder had different types of fasting including fasting including avoidance of only ASF or ASF excluding fish (Fig. 1).

Analysis of the difference in lipid profiles, blood sugar and measures of body composition between fasters of ASF who skipped breakfast up to 12:00 AM and those who skipped breakfast until 15:00 PM showed that the outcome measures were better in the fasters up to 15:00 Pm. However, there was no statistically significant difference except in waist circumference. In the case of waist circumference there was a significantly lower value among fasters up to 15:00 PM (P = 0.027).

**Figure 2:**
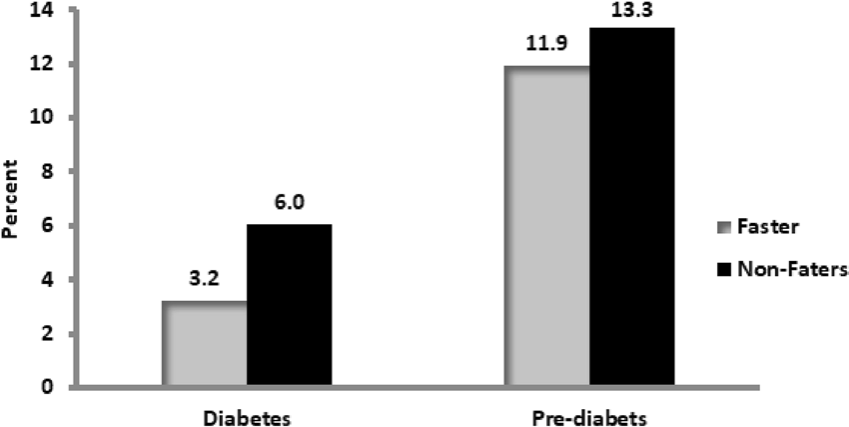
Diabetes and prediabetes among fasting and non-fasting employees of Jimma University, Southwest Ethiopia.

As presented in Fig. 2, analyses of the proportion of diabetic and pre-diabetic people by fasting status showed that fasters had lower prevalence of both conditions compared to non-fasters although the deference was not statistically significant(P = 0.098).

### Predictors of body composition, blood sugar and lipid profiles

On multivariable linear regression analyses, after adjusting for different variables, fasting had significant association with low FBS, Low Density Lipoprotein (LDL), T.chol and TG levels and higher level of high Density Lipoprotein (HDL). Fasters had higher levels of HDL by 6.9 mg/dl (β = 6.9, *P* < *0.05*), while they had lower levels of T.chol by 8.5 mg/dl (β = −8.5, *P* < *0.05*), TG by 14.3 mg/dl (β = −14.3, P < 0.05), FBS by 8.6 mg/dl (β = −8.6, *P* < *0.05*) and LDL by 10.3 mg/dl (β = −10.3, *P* < *0.01*).

Other variables which were significantly associated with high level of HDL were age and hours of sleep in a day. A one hour increase in the duration of sleep was associated with higher HDL level by 6.7 mg/dl (β = 6.7, *P* < *0.001*) and lower LDL level by 1.2 mg/dl (β = −1.2, *P* < *0.05*).

Similarly, a year increase in age was associated with higher levels of: T.chol level by 1.5 mg/dl (β = 1.5, P < 0.001), Triglycerides by 2.1 mg/dl (β = 2.1, *P* < *0.001*), FBS by 0.5 mg/dl (β = 0.5, P < 0.05) and LDL by 0.6 mg/dl (β = 0.6, *P* < *0.01*). Being in the highest tertiles of sitting hours was associated with higher level of LDL by 8.6 mg/dl (β = 8.6, *P* < *0.05*). Sex was another independent predictor of lipid profiles. Males had lower LDL and T.chol by 15 mg/dl (β = −15, *P* < *0.001*) and 12.9 mg/dl (β = −12.9, *P* < *0.01*), respectively, while they had higher Triglyceride level by 20.1 mg/dl (β = 20.1, *P* < *0.01*) compared to females (Table 3).

**Table 3:** Multivariable linear regression models predicting fasting blood sugar and lipid profiles among workers of Jimma University.

Predictors	HDL	T.chol	TG	FBS	LDL
	β (95%CI)	β (95%CI)	β (95%CI)	β (95%CI)	β (95%CI)
Sex					
Male	4.6 (−1.7, 10.8)	−14.5 (−22.5,−6.5)***	16.1 (2.3, 29.9)*	5.5 (−2.1, 13.2)	−18.2 (−25.7, −10.8)***
Female (Reference)					
Age (Yrs)	0.0 (−0.3, 0.3)	1.5 (1.1, 1.9)***	2.1 (1.5, 2.8)***	0.5 (0.1, 0.8)*	0.6 (0.2, 0.9)**
ASF Fasting and breakfast skipping					
Yes	6.9 (1.2, 12.7)*	−8.5 (−15.9, −1.1)*	−14.3 (−27.0, −1.6)*	−8.6 (−15.7, −1.6)*	−10.3 (−17.2, −3.4)**
No (Reference)					
Days of moderate exercise per week	−2.2 (−9.0, 4.6)	2.1 (−6.6, 10.9)	−3.2 (−18.2, 11.8)	−3.2 (−11.5, 5.1)	3.7 (−4.4, 11.8)
Tertiles of hours spent sitting in a day					
Long	3.2 (−10.1, 3.8)	−1.0 (−9.9, 8.0)	0.7 (−14.6, 16.0)	6.4 (−2.1, 14.9)	8.6 (0.3, 16.9)*
Medium	−4.0 (−10.4, 2.4)	−2.9 (−11.2, 5.3)	−7.2 (−21.3, 6.9)	6.5 (−1.3, 14.4)	2.1 (−5.5, 9.8)
Short (Reference)					
Hours of sleep per day	6.7 (6.0, 7.5)***	−0.4 (−1.4, 0.6)	−0.5 (−2.2, 1.2)	−0.3 (−1.3, 0.6)	−1.2 (−2.1, −0.2)*
Educational status					
High school and below (Reference)	−4.5 (−12.0, 3.0)	7.3 (−2.3, 17.0)	5.6 (−10.9, 22.1)	0.8 (−8.3, 10.0)	
Diploma	−2.7 (−12.2, 6.7)	2.9 (−9.3, 15.1)	16.3 (−4.6, 37.2)	11.3 (−0.4, 22.9)	−0.8 (−9.7, 8.1)
Degree and above					−7.2 (−18.5, 4.2)
Type of Employee					
Academic staff	2.9 (−11.2, 5.4)	4.2 (−6.4, 14.9)	1.1 (−17.2, 19.4)	−6.8 (−16.9, 3.4)	4.7 (−5.1, 14.6)
Hospital technical staf	2.0 (−11.1, 15.2)	−1.7 (−18.6, 15.3)	−15.7 (−44.7, 13.4)	−7.7 (−23.8, 8.4)	−6.7 (−22.4, 9.0)
Admin staff (Reference)					
Monthly Income Tertile					
High	−1.7 (−11.1, 7.7)	9.6 (−2.4, 21.7)	−6.3 (−27.0, 14.4)	−1.5 (−12.9, 10.0)	5.3 (−6.0, 16.5)
Medium	−4.5 (−11.6, 2.6)	0.8 (−8.3, 9.9)	−6.8 (−22.4, 8.8)	−1.5 (−10.2, 7.2)	3.3 (−5.2, 11.8)
Low (Reference)					
Frequency of alcohol drinking					
1–3 times per month or more frequently	−3.8 (−10.4, 2.9)	3.6 (−4.9, 12.1)	14.2 (−0.4, 28.9)	6.5 (−1.6, 14.7)	1.2 (−6.7, 9.2)
Non-drinker or less than once per month (Reference)					

***P < 0.001, **P < 0.01, *P < 0.05.

HDL = High density lipoprotein, LDL = low density Lipoprotein, FBS = Fasting blood Sugar, T.chol = Total Cholesterol,

Maximum VIF for all models <3.

A second multivariable linear regression analyses of different body composition parameters showed that fasters had lower WC by 2.4 cm (β = −2.4*, P* < *0.01*) and lower body fat percent by 3.0 (β = −3.0, *P* < *0.001*). Another variable associated with body composition was sex. Males had lower BMI and body fat percent by 3.5 kg/m^2^ (β = −3.5, *P* < *0.001*) and 15.5 (β = −15.5, P < 0.001), respectively compared to females.

Conversely, as age increased by one year WC, BMI and body fat percent increased by 0.4 cm (β = 0.4, *P* < *0.001*), 0.1 kg/m^2^ (β = 0.1, *P* < *0.001*) and 0.3(β = 0.3, *P* < *0.001*), respectively. Study participants in the higher and middle monthly income tertiles had higher WC by 5.3 cm (β = 5.3, *P* < *0.001*) and 2.5 cm (β = 2.5, *P* < *0.05*), respectively. They also had higher BMI by 1.9 kg/m^2^ (β = 1.9, *P* < *0.01*) and 0.9 kg/m^2^ (β = 0.9, *P* < *0.05*), respectively. There was also a higher body fat percent for the high income tertile by 2.9 (β = 2.9, *P* < *0.01*). Participants who drank alcohol 1–3 days or more frequently per month had high WC by 2.5 cm (β = 2.5, *P* < *0.05*) compared to non-drinkers (Table 4).

**Table 4:** Multivariable linear regression model predicting Body mass index, waist circumference and body fat percent among workers of Jimma University.

Predictors	WC	BMI	BF%
	β (95%CI)	β (95%CI)	β (95%CI)
Sex			
Male	−1.4 (−3.2, 0.4)	−3.5 (−4.3, −2.7)***	−15.5 (−17.0, −14.0)***
Female (Reference)			
Age (Yrs.)	0.4 (0.3, 0.5)***	0.1 (0.1, 0.2)***	0.3 (0.2, 0.3)***
Fasting ASF and breakfast skipping			
Yes	−2.4 (−4.0, −0.8)**	−0.2 (−0.9, 0.5)	−3.0 (−4.4, −1.7)***
No (Reference)			
Days of moderate exercise per week	−0.3 (−2.2, 1.7)	0.2 (−0.6, 1.0)	0.1 (−1.5, 1.7)
Hours spent sitting in a day tertiles			
Long	0.6 (−1.4, 2.6)	0.7 (−0.1, 1.6)	0.6 (−1.0, 2.2)
Medium	0.1 (−1.7, 1.9)	0.1 (−0.7, 0.9)	−0.3 (−1.8, 1.2)
Short (Reference)			
Hours of sleep per day	−0.1 (−0.3, 0.1)	0.0 (−0.1, 0.1)	−0.1 (−0.3, 0.1)
Educational status			
High school and below (Reference)	0.3 (−1.9, 2.4)	0.4 (−0.6, 1.3)	0.0 (−1.7, 1.8)
Diploma	0.6 (−2.1, 3.3)	0.4 (−0.8, 1.5)	1.2 (−1.0, 3.4)
Degree and above			
Type of Employee			
Academic staff	0.0 (−2.3, 2.4)		−0.6 (−2.6, 1.3)
Hospital technical staff	−1.6 (−5.4, 2.1)	0.2 (−0.8, 1.2)	0.2 (−2.9, 3.3)
Administrative staff (Reference)		0.4 (−1.2, 2.1)	
Monthly Income Tertile			
High	5.3 (2.6, 7.9)***		2.9 (0.7, 5.1)**
Medium	2.5 (0.5, 4.5)*	1.9 (0.7, 3.0)**	1.5 (−0.1, 3.2)
Low (Reference)		0.9 (0.1, 1.8)*	
Frequency of drinking alcohol	2.5 (0.6, 4.4)**	0.7 (−0.2, 1.5)	−1.0 (−2.6, 0.5)
1–3 days per month or More frequently			
Non-drinker or less than once per month (Reference)			

***P < 0.001, **P < 0.01, *P < 0.05.

WC = Waist Circumference, BF% = Body fat percent. BMI = body mass index.

Maximum VIF for all models <3.

## Discussion

We found out that breakfast skipping and animal source food avoidance  was significantly associated with lower mean TG, T.chol, FBS and LDL and a higher mean HDL. There was also a significantly low mean WC and body fat percent among fasters. These findings are consistent with studies elsewhere^3,4,15,19,20^.

Evidence shows that vegetarian diet and intermittent fasting were effective in reducing the risk of metabolic syndrome, its components and other lipid profiles^8,12,14,21^. A study on one afternoon meal per day for a duration of about two months demonstrated reductions in fasting glucose and improvements in LDL and HDL^19^. Another study showed that breakfast skipping was effective in favorably improving selected metabolic parameters and body weight^15^. Intermittent fasting was suggested to be a dietary method that can improve lipid profiles in healthy, obese and dyslipidemic men and women by reducing total cholesterol, LDL, TG and increasing HDL levels^20^.

So far, the effect of intermittent fasting on the lipid profile and body weight loss was determined based on Ramadan fasting^20^, indicating that the investigation of “Hudade” fasting practices on lipid profile and body composition done in this study will add to the existing body of evidence. The benefit of fasting in reducing total cholesterol, weight, blood sugar, and improving insulin sensitivity were reported from restricted mice feeding^19^.

Another component of fasting in this study was avoiding consumption of animal source foods by the fasters. A review of evidence shows that vegetarian diets were effective in lowering serum levels of HDL, total cholesterol and LDL^22^, and in improving lipid metabolism by reducing the body mass index (BMI)^23^. Moreover, the protective effect of vegetarian diet through lowering the risk for diabetes; but not for metabolic syndrome and obesity was also implicated^24^. Consumption of animal fats on the other hand leads to increased risk of metabolic syndrome as saturated fats from animals and trans fats are indicated to be the main causes of metabolic syndrome^12^. High intake of animal protein and fat and low fiber is known to increase the risk of different organ diseases including cardiovascular diseases^25^ and Chronic Kidney Diseases^26^. It was indicated that vegetarian diets could be useful non-pharmacological means of managing dyslipidemia, especially hypercholesterolemia^22^.

Fasting could generate durable health effects through several pathways^2,4^. Intermittent fasting is the most feasible intervention that exerts a protective effect on cardiovascular diseases^2^, obesity, hypertension, asthma, and rheumatoid arthritis^3^. The protective effect of intermittent or periodic fasting from degenerative diseases such as cancers, heart disease, diabetes and neurological problems and aging has been reported from animal studies^3^.

Intermittent fasting is hypothesized to exert its protective effect against metabolic disorders through its effects on circadian biology, microbiome of the gut and sleep pattern^3,4^. Evidence shows that even a single overnight fasting can reduce serum levels of multiple biomarkers of chronic diseases^4^. A study on obese individuals showed that intermittent fasting reduces oxidative stress^2^ indicating that fasting animal source foods may reduce the risk of metabolic syndrome. This could be due to the fact that fasting induces a negative energy balance leading to utilization of excess body fat for energy production^27^. In lower eukaryotes, chronic fasting extends longevity, in part, by reprogramming metabolic and stress resistance pathways^3^. Thus, fasting has the potential to delay aging and help to prevent and treat diseases while minimizing the side effects caused by chronic dietary interventions^3,28^. Fasting could offer a potentially promising preventive strategy for improving health at the population level, with multiple public health benefits^4^.

There have been several dietary strategies to prevent and treat metabolic syndrome and associated chronic non-communicable diseases. The Mediterranean dietary pattern, omega 3 fatty acids, antioxidant intake, negative-energy-balance diets and reducing meal frequency have been used as effective strategies to prevent and treat metabolic syndrome^29^. In this study, desirable effect of animal source food avoiding  and breakfast skipping related to religious practice were documented.

It was also observed that age, sex, sleep hours, alcohol drinking and income were important predictors of lipid profiles and body composition. In this study, women had higher levels of lipid profile parameters and body fat percent compared to males. This could be due to the difference in sex hormone levels^30^. There was also a significant positive association between age and LDL, TG, T.chol, BMI, waist circumference and body fat, which is consistent with other, studies^30,31^. Body fat percent appears to increase significantly after the age of 40 years due to weight gain^32^.

We also found out that alcohol drinking was positively associated with waist circumference, which could be due to the fact that alcohol increases central obesity as it contributes to the energy pool of the body^33,34^. There was also a significant positive association between sleep hours and HDL level and negative association with LDL level. High HDL and low LDL levels were reported among people usually sleeping for eight or more hours per day^35^. This may be due to the fact that sleep has a strong influence on the metabolic hormones. Sleep restriction lowers appetite suppressing hormone (leptin) and increases appetite promoting hormone (ghrelin), leading to high dyslipidemia including low HDL and high LDL^36^. Similarly, the highest tertile of sitting hours was associated with increased LDL level by 8.6 mg/dl, which is consistent with the finding of another study that demonstrated a positive association between triglycerides, LDL, and total cholesterol with all sedentary behavior^37^.

The findings of this study have a strong practical implication; especially in the context of the newly developed national food and nutrition policy of Ethiopia and aspiration of the country to be in the lower middle income category by 2025. Economic growth compounded with globalization, rapid urbanization, motorized way of life and increasing life expectancy fuels a rapid emergence of chronic non-communicable diseases. This study demonstrated the, great potential that ASF fasting and breakfast skipping could have in addressing sustainable development goals^38,39^ through averting risks of emerging problems and the toll of associated morbidity and mortality. The development of food and nutrition strategy should consider ways of enhancing such dietary modification strategies.

In this study, we acknowledge the following limitations. Although ASF fasting and breakfast skipping were shown to have desirable effects on lipid profiles and body composition, the sustainability of such behaviors when done in non-fasting periods and their effect on micronutrient status is not known. Moreover; we were not able to differentiate whether or not the observed changes reported in this study are due to, breakfast skipping or elimination of animal products or a combination of both. Future research is needed to unpack the effects. Having a baseline data on the outcomes would have helped to determine the difference of the differences. However, given that the outcomes are health problems or risk factors for health problems, it was not ethically feasible to do the baseline data and leave them without intervention. A retrospective cohort design was employed, and we adjusted in the model for background characteristics that could potentially confound the results. Future research should address these limitations. It is also possible that presence of diabetes can affect an individual’s decision on whether or not to fast, which could affect the prevalence of diabetic individuals in the non-fasting group. To overcome this, known cases of diabetes and hypertension were excluded from the study as they were taking medications which may interfere with their fasting status.

Moreover, our analyses showed that there was no a significant difference (P = 0.098) in the proportion of diabetic people between fasters (3.2%) and non-fasters (6.0%), making this possibility thin.

## Conclusion

Animal source food fasting and breakfast skipping has a significant desirable health effects on lipid profiles, fasting blood sugar and body composition. The findings imply that such a dietary practice could be considered as a basis for public health promotion. Future research should look at the effect of such a practice on micronutrient intake and investigate the sustainability of metabolic changes and their long term effects after cessation of fasting. Moreover, the effect of such a stringent fasting practice on micronutrient status and the minimum number of days of fasting to generate the observed effects should be investigated.

## Data Availability

The datasets used and/or analysed during the current study are available from the corresponding author on reasonable request.
